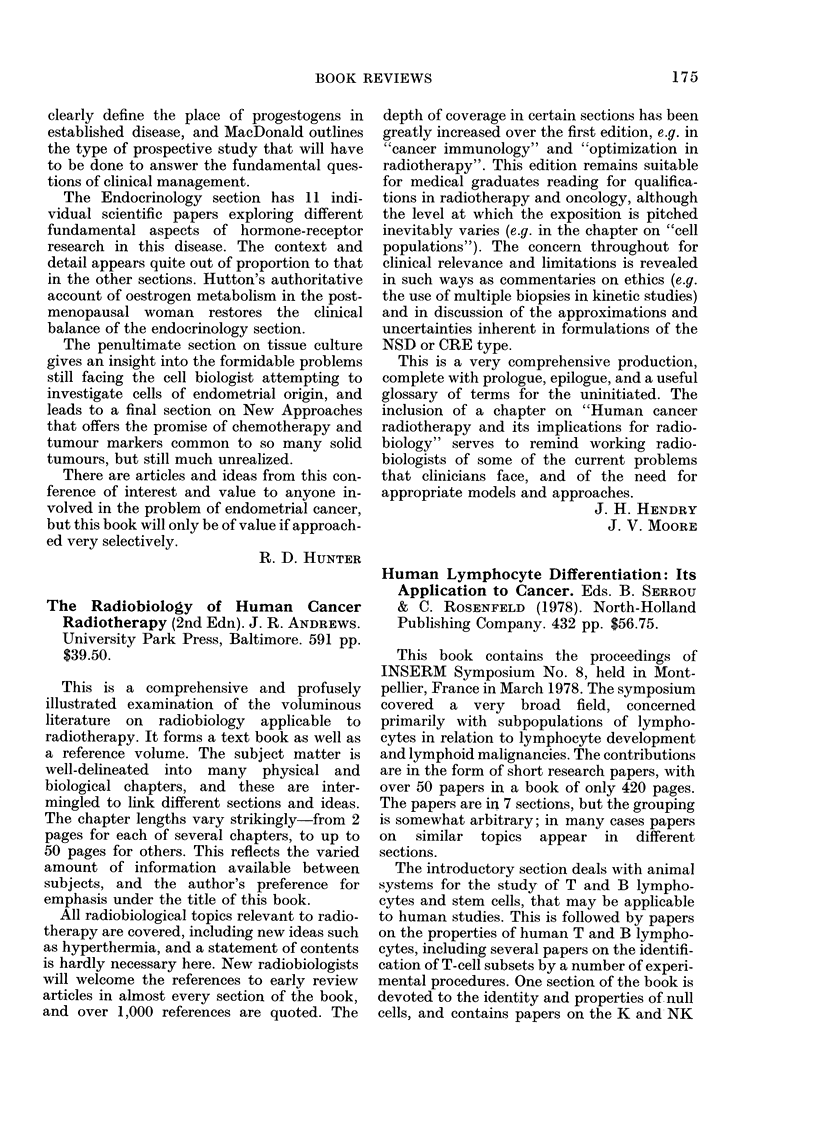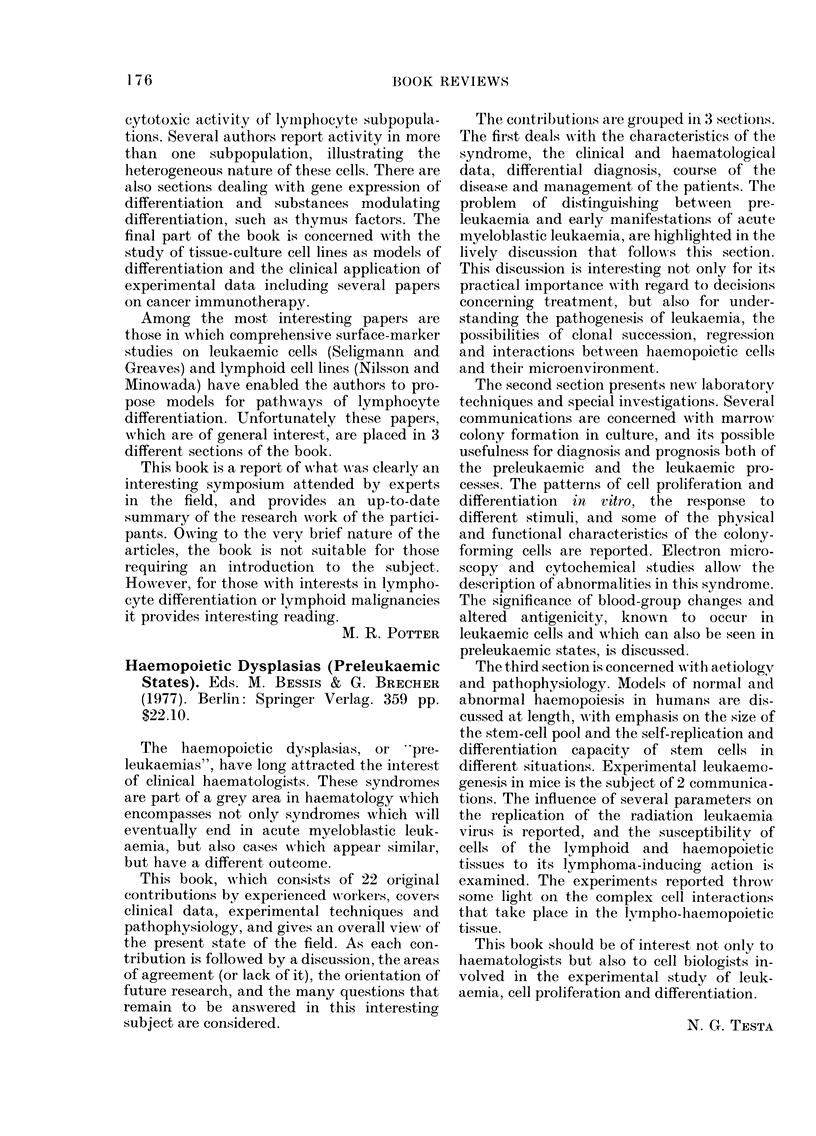# Human Lymphocyte Differentiation: Its Application to Cancer

**Published:** 1979-07

**Authors:** M. R. Potter


**Human Lymphocyte Differentiation: Its Application to Cancer.** Eds. B. SERROU & C. ROSENFELD (1978). North-Holland Publishing Company. 432 pp. $56.75.

This book contains the proceedings of INSERM Symposium No. 8, held in Montpellier, France in March 1978. The symposium covered a very broad field, concerned primarily with subpopulations of lymphocytes in relation to lymphocyte development and lymphoid malignancies. The contributions are in the form of short research papers, with over 50 papers in a book of only 420 pages. The papers are in 7 sections, but the grouping is somewhat arbitrary; in many cases papers on similar topics appear in different sections.

The introductory section deals with animal systems for the study of T and B lymphocytes and stem cells, that may be applicable to human studies. This is followed by papers on the properties of human T and B lymphocytes, including several papers on the identification of T-cell subsets by a number of experimental procedures. One section of the book is devoted to the identity and properties of null cells, and contains papers on the K and NK

cytotoxic activity of lymphocyte subpopulations. Several authors report activity in more than one subpopulation, illustrating the heterogeneous nature of these cells. There are also sections dealing with gene expression of differentiation and substances modulating differentiation, such as thymus factors. The final part of the book is concerned with the study of tissue-culture cell lines as models of differentiation and the clinical application of experimental data including several papers on cancer immunotherapy.

Among the most interesting papers are those in which comprehensive surface-marker studies on leukaemic cells (Seligmann and Greaves) and lymphoid cell lines (Nilsson and Minowada) have enabled the authors to propose models for pathways of lymphocyte differentiation. Unfortunately these papers, which are of general interest, are placed in 3 different sections of the book.

This book is a report of what was clearly an interesting symposium attended by experts in the field, and provides an up-to-date summary of the research work of the participants. Owing to the very brief nature of the articles, the book is not suitable for those requiring an introduction to the subject. However, for those with interests in lymphocyte differentiation or lymphoid malignancies it provides interesting reading.

M. R. Potter